# Transforming Diabetes Management in Rural America: A Qualitative Exploration of a Diabetes Coaching Program Delivered via Telehealth

**DOI:** 10.3390/ijerph23060696

**Published:** 2026-05-25

**Authors:** Catherine Moring, Caroline Brock, Katharine L. Brown, Allison Ford-Wade

**Affiliations:** 1 Tallahatchie General Hospital, James C. Kennedy Wellness Center, Charleston, MS 38921, USA; 2 Department of Public Health, University of Mississippi, Oxford, MS 38677, USA; cbrock@go.olemiss.edu (C.B.); ford@olemiss.edu (A.F.-W.); 3 Department of Criminology and Criminal Justice, Indiana University, Bloomington, IN 47405, USA; klb22@iu.edu

**Keywords:** diabetes, telehealth, self-management, qualitative research, rural health, rural health services, chronic disease, health planning

## Abstract

**Highlights:**

**Public health relevance—How does this work relate to a public health issue?**

- Diabetes is a major public health issue in rural and underserved communities, where barriers such as limited healthcare access, transportation challenges, food insecurity, and provider shortages make effective self-management more difficult.
- This study examines a telehealth-based Diabetes Self-Management Education and Support program designed to improve access to individualized diabetes coaching for rural adults living with diabetes or prediabetes.

**Public health significance—Why is this work of significance to public health?**

- Findings highlight the value of personalized, relationship-centered telehealth coaching in improving diabetes knowledge, self-efficacy, lifestyle behaviors, and perceived quality of life among rural participants.
- The study adds patient-centered qualitative evidence to the growing literature on telehealth DSMES, demonstrating that accessible coaching can help address rural diabetes disparities beyond clinical outcomes alone.

**Public health implications—What are the key implications or messages for practitioners, policy makers and/or researchers in public health?**

- Practitioners should consider telehealth DSMES models that combine practical education, individualized goal setting, emotional support, and accountability to promote sustainable diabetes self-management.
- Policymakers and payers should strengthen reimbursement pathways for telehealth-based DSMES so evidence-informed programs can be scaled and sustained in rural and medically underserved communities.

**Abstract:**

Diabetes disproportionately affects rural populations in the United States where prevalence and associated complications remain among the highest in the nation. Access to diabetes education and support services is often limited by geography, socioeconomic barriers, and workforce shortages. This study qualitatively explored participant experiences in a telehealth-based Diabetes Self-Management Education and Support (DSMES) program. This study uses interview techniques and takes a phenomenological approach to exploring the lived experiences of 27 program participants. Transcripts were analyzed through three cycles of coding to identify shared themes. Four themes emerged: (1) structural benefits of the program, (2) knowledge gained, (3) lifestyle changes implemented, and (4) improved quality of life. Participants consistently emphasized the value of personalized coaching, emotional encouragement, and practical nutrition education. Findings suggest that individualized telehealth coaching plays an important role in diabetes self-management, particularly in rural and underserved populations. By combining personalization with education and encouragement, programs can improve patient engagement, enhance self-efficacy, and support meaningful behavior change.

## 1. Introduction

Diabetes remains one of the most pressing public health challenges of the 21st century, with global prevalence steadily increasing over the past three decades. In the United States, more than 37 million adults live with diabetes, and an estimated one in five are undiagnosed [1]. Mississippi consistently ranks among the states with the highest diabetes prevalence, with approximately one in seven adults diagnosed and disproportionate morbidity and mortality compared to national averages [2]. Mississippi’s high diabetes burden reflects a convergence of structural and social determinants of health shaped by high levels of rurality, poverty, obesity, physical inactivity, limited access to primary and specialty care, and challenges accessing affordable healthy foods [3]. These factors are particularly pronounced in rural areas such as the Mississippi Delta, where communities often face medical underservice, transportation barriers, food-access limitations, and fewer opportunities for preventive care and diabetes self-management support. Thus, Mississippi serves as an important example of how diabetes prevalence is shaped not only by individual risk factors, but also by broader geographic, economic, and health-system conditions that may be relevant to other rural and medically underserved settings.

These trends place an enormous strain on healthcare systems, with direct medical costs in Mississippi alone estimated at $2.4 billion annually [4]. The burden of diabetes is exacerbated in rural regions, where barriers such as limited healthcare infrastructure, geographic isolation, socioeconomic disadvantage, and high rates of obesity complicate disease management. In such contexts, access to diabetes education and self-management support is particularly scarce. Yet, effective diabetes self-management education and support (DSMES) is critical for reducing complications, improving glycemic control, and empowering individuals to engage in sustainable lifestyle changes.

Telehealth has emerged as a promising solution to address gaps in chronic disease management [5,6]. By leveraging phone, video, and digital platforms, telehealth-based DSMES can reduce barriers related to transportation, time, and access to qualified providers. Evidence suggests that telehealth and digital DSMES interventions can improve diabetes self-management and produce outcomes comparable to traditional in-person education [7]. Recent systematic reviews have found that online DSMES can achieve biomedical outcomes comparable to face-to-face education, while digital interventions that include timely, personalized coaching may be particularly effective in supporting glycemic improvement and patient engagement [5,6]. In rural settings, telemedicine-based DSMES has also been shown to be acceptable to patients and capable of producing A1c improvements similar to in-person DSMES. However, fewer studies have qualitatively examined how participants experience individualized telehealth DSMES coaching, particularly in rural and underserved communities.

By documenting participant voices, the present study adds qualitative depth to the growing telehealth DSMES literature and provides practical insights into how personalized coaching, accessible delivery, emotional support, and patient-centered education may support engagement, self-efficacy, lifestyle change, and quality of life among rural adults living with diabetes. Diabetes Solutions, an accredited DSMES program, seeks to bridge a gap by providing personalized coaching through telehealth delivered to participants living in rural areas. Delivered by a multidisciplinary team, including Certified Diabetes Care and Education Specialists (CDCES), a registered nurse, a registered dietitian, and a health coach, the program tailors the frequency and focus of sessions to individual participant needs.

Using a phenomenological approach, the present study qualitatively explores the lived experiences of participants enrolled in the Diabetes Solutions program to better understand how participants perceive and experience this model of care. Specifically, this study asks one core question: How do individuals perceive and make meaning of their lived experiences with the Diabetes Solutions telehealth coaching program? In doing so, this study provides directions for practitioners on increasing accessibility to healthcare via telehealth programming, specifically for individuals living with prediabetes or diabetes in rural areas. By documenting the lived experiences of patients, this study contributes to the growing body of literature on telehealth and DSMES while providing directions for improving participant experiences with telehealth interventions in rural populations.

## 2. Methods

### 2.1. Program Description

Diabetes Solutions is an accredited DSMES program designed to provide individualized coaching through telehealth modalities, including telephone and Zoom. Frequency of sessions varied by participant preference and need, ranging from weekly to quarterly, with an average session number of four and average length of approximately 30 min with the exception of the first and sometimes second session which were closer to an hour.

Program participants receive an average of four to five coaching sessions within the first six months of enrollment. Many participants complete their coaching within six months while others choose to stay enrolled and receive monthly coaching sessions over a year as well as quarterly coaching sessions after their first year of enrollment. For Medicare beneficiaries, Medicare providers’ reimbursement for two hours of coaching for all subsequent years following year one. The program delivery and design allow for modification to ensure participants’ needs are met while also reaching treatment goals. In addition to coaching, all program participants are seen by a primary care provider or endocrinologist regularly for their clinical needs such as medication refills and referrals to specialty care.

### 2.2. Methodology

The present study adopts a constructivist worldview. Constructivism argues that multiple realities can exist, or put simply, that there are different ways individuals can make meaning of the world around them [8,9]. This is in opposition to a positivist framework, which underscores the importance of objective reality and measurable truths, lending itself towards causal research designs (i.e., hypothesis testing). Constructivism is suitable for qualitative research for several reasons. First, the goals of qualitative research are to provide rich text describing how individuals make meaning of their experiences in natural settings, underscoring the importance of subjective realities in place [8]. Naturally, a qualitative approach ensures the centering of subjectivity in meaning-making, as well as the co-construction of knowledge between researchers and participants, which are core strategies adopted in this study. Second, there is no assumption of statistical generalizability in qualitative research, rather the findings of qualitative research produce naturalistic (i.e., the connection between research findings and the lived experiences of the reader) and inferential generalizability (i.e., results can be transferrable to other settings) [10]. This supports the assumption that the lived experiences individuals have with the world around them can vary, yet they may have a degree of transferability to other settings (e.g., individuals living with diabetes in similarly situated contexts). Ultimately, adopting a constructivist approach to the current research question allows the researcher and participants to co-construct how individuals living with prediabetes or diabetes make meaning of their experiences with healthcare options, specifically Diabetes Solutions.

The current research question lends itself well to a phenomenological design (i.e., research designs aimed at identifying common meaning among a group of individuals with a shared phenomenon for several reasons [8,11]. First, participants all share a similar phenomenon, being enrolled in Diabetes Solutions. Second, the research question for this study highlights the importance of lived experiences. Phenomenological studies seek to understand lived experiences, specifically shared lived experiences. Diabetes Solutions provides an opportunity to explore how participants experience the program individually, but also how those lived experiences are shared among participants.

### 2.3. Participant Selection

The present study uses purposeful sampling strategies to recruit participants, which is consistent with recommendations for a phenomenological design [8]. In Spring of 2025 participants were recruited via the Diabetes Solutions team. Participants were selected from the pool of individuals who participated in the program between 1 January 2023, and 31 December 2024. To ensure that interview participants had sufficient exposure to the intervention and adequate clinical data for context, potential participants were eligible for this qualitative study if they had at least two A1c measurements during the study period and had completed at least four coaching sessions. Additional eligibility criteria included a diagnosis of diabetes or prediabetes, being aged 18 or older, and consenting to participate in an interview.

From the eligible pool of 146 participants, 30 individuals were purposively selected and invited to participate in an interview. Selection was guided by the goal of obtaining information-rich interviews from participants who had sufficient exposure to the intervention and could provide detailed reflections on program experiences, barriers, facilitators, and perceived outcomes. During selection, efforts were made to include participants with a range of clinical responses, rather than only those with significant A1c reductions or other favorable health outcomes. Of the 30 invited, 27 consented and completed interviews. Demographic characteristics of the 27 participants are presented in Table 1.

The target sample size was established a priori with the goal of interviewing at least 20 participants, which is consistent with qualitative research literature suggesting that thematic saturation can often be reached with relatively small, focused samples, particularly when the study population is relatively homogeneous, and the research questions are narrowly defined. Importantly, 27 participants is consistent with prior phenomenological research [12] and recommended sample sizes for phenomenological studies (between 5 and 250) [8,13]. A recent systematic review found that saturation in interview-based qualitative studies is commonly achieved within approximately 9 to 17 interviews, while earlier empirical work by Guest and colleagues found that major themes often emerge within the first 12 interviews [14,15]. Therefore, the final sample of 27 participants was considered adequate to capture a range of participant experiences while remaining consistent with accepted standards for qualitative inquiry.

One member of the research team conducted one-on-one structured interviews with participants using a 14-question guide covering enrollment motivations, telehealth experiences, knowledge gained, lifestyle changes, health outcomes, and program perceptions. The full list of interview questions is presented in Appendix A (Table A1). Interviews were conducted via phone and lasted approximately 30 min. All interviews were recorded and later transcribed into naturalized transcripts (i.e., maintained filler language such as “um” or “uh” to capture the essence of participant responses). Structured interviews provide a means of collecting data on individual experiences with Diabetes Solutions while also keeping participants focused on the specific phenomenon. While unstructured and semi-structured interviews would afford more opportunities to co-construct knowledge and capture deep reflection through probing questions, structured interviews can still provide rich text that captures lived experiences. Moreover, phenomenological methodologists underscore the importance of focusing on the lived experience rather than the means by which it is captured [16] and underscore the importance of active listening throughout interviews, which can be impacted if probing questions “go on a tangent out of curiosity” [17] (p. 7) or to address discomfort through conversation. Structured interviews provide participants opportunities to share without questions that could potentially distract from their lived experiences. Participants received a $50 Visa gift card as compensation. All procedures were approved by the University of Mississippi’s Institutional Review Board prior to beginning data collection.

### 2.4. Analytic Strategy

Consistent with coding recommendations by Saldaña, data were analyzed across three cycles of coding [18]. While phenomenological studies often take a less structured approach to analysis (e.g., interpretive phenomenology), without a structured coding process, the size of our sample may “suppress the voices of participants and descriptions of phenomena,” [16] (p. 9) resulting in lower quality research findings. Thus, we employ structured coding techniques with a phenomenological approach to capture the lived experiences of participants with Diabetes Solutions, thus reducing the inclination for participant responses to overshadow one another during interpretation. In the first cycle of coding, descriptive codes were applied to capture the initial lived experiences of each participant. In this cycle, the coder read and re-read the transcripts several times to have comprehensive descriptive codes across participants. The second cycle of coding involved collapsing codes into common themes that captured shared experiences among participants. In the final coding cycle, themes were synthesized to reflect shared experiences with Diabetes Solutions. Importantly, coding in cycles is a flexible coding strategy that can be adopted for many epistemological approaches, including phenomenology [18], by centering the assumptions of the approach. Indeed, for the present study, codes and themes were developed in the spirit of identifying the essence of lived experiences among telehealth program participants and how those themes were shared among participants. All coding was conducted in ATLAS.ti 26.

Creswell & Poth recommend researchers adopt two strategies to manage bias and ensure trustworthiness when analyzing qualitative data [8]. This study adopts four core strategies to ensure trustworthiness: (1) reflexivity, (2) memoing, (3) triangulation and (4) peer debriefing to create an audit trail detailing the coding process. Reflexivity, not to be confused with reflexive thematic analysis, is the process of actively reflecting on positionality during the coding and interpretation processes. Reflexivity ensures researchers are “giving place to ethical considerations within one’s research practice [which] can provide more insightful research findings” [19] (p. 285); see also [20]. Importantly, reflexivity is recommended for scholarship in the health sciences when conducting qualitative research [21]. In addition to reflexivity, the creation of an audit trail via active “memoing” during the coding and interpretation processes occurred. The main coder actively detailed coding decisions as well as ongoing introspection prompted by reflexivity, enhancing opportunities to be aware of biases and personal reactions during the coding and interpretation processes.

Consistent with recommendations by Lietz et al., the research team also coupled triangulation and peer-debriefing strategies to identify themes capturing the lived experiences of research participants [8,20,22]. Triangulation is a strategy wherein members of the research team discuss emergent themes and patterns in the data and how they should be interpreted. In practice, this involved all co-authors discussing the themes in the data and what they were describing in terms of meaning-making and the lived experiences of participants with Diabetes Solutions. Peer debriefing involves conversations regarding the coding process and the identification of themes between the research team and an impartial colleague. The third author on this manuscript serves as the core individual used for peer-debriefing, as they were not involved in the development, implementation, or data collection processes for Diabetes Solutions, but specialize in qualitative methodology in an entirely different field. Peer debriefing between the first and third authors occurred throughout each cycle of the coding process as well as during the interpretation of emergent themes. Debriefing continued between the third author and the research team at large during the writing of the results and discussion. This was especially important because coding was conducted by one member on the research team; thus, triangulation and peer-debriefing provided opportunities for all members of the research team to discuss codes throughout the coding process and the development of them into emergent themes capturing the essence of participant experiences. The analytic strategy outlined in this study is consistent with qualitative and phenomenological research designs across multiple disciplines [23,24,25,26,27,28]. Four central themes emerged from the analysis.

## 3. Results

Participants ranged in age from 21 to 75 years, with the majority (55.6%) aged 60–79 years. Most participants were female (77.8%). The sample included individuals identifying as White (59.3%) and Black (40.7%). Two (7.5%) participants had a diagnosis of Pre-diabetes, and 25 (92.5%) participants had a diagnosis of Type 2 Diabetes. The average number of sessions completed by participants was 8.5 and ranged from 4 to 15 sessions. These characteristics provide important context for understanding the lived experiences represented in the findings.

The overarching essence of participant experiences with Diabetes Solutions underscored the importance of individualized healthcare for empowering practical and sustainable diabetes management. This shared essence was prompted by experiences with: (1) program structure, (2) knowledge attainment, (3) lifestyle changes, and (4) quality of life changes. The names used below do not reflect the participants’ real names but are representative of their gender to help the reader feel a sense of connection to the participants’ lived experience.

### 3.1. Theme 1: Experiences with Program Structure

Experiences with the individualized structure of Diabetes Solutions prompted participants to feel that they had a healthcare provider who made concerted efforts to see them and understand their needs. This experience prompted participants to feel safe enough to be vulnerable with their provider by sharing aspects of their health they may not have otherwise been inclined to share. Participants highlighted the importance of the personalized nature of the program, which made space for encouragement from coaches and accountability provided during regular check-ins. Many emphasized that coaching felt tailored to their individual mindset, habits, and relationships with food. One participant noted:

“*I feel like I could talk to her [coach] about anything related to my health. She was never condescending. She would just say, ‘It’s a journey, these are the tools you need.’ She was a cheerleader in my corner, and that really worked for me.*” (Jessica, 67)

In the above quote, the participant underscores the importance of individualized programming for creating experiences that allowed them to actively discuss their health without judgement. In doing so, this provided additional opportunities for their healthcare provider to identify the specific tools needed to manage their diabetes. Echoing this, another participant noted how their experiences with individualized programming differed in that it did not feel like a “cookie-cutter” approach.

“*It wasn’t like this cookie-cutter, step one, step two. She knows my mindset, my relationship with food. We were able to create a game plan that worked for me.*” (Ashley, 30)

In essence, participant experiences with individualized healthcare illustrate the importance of openness from providers to hear and understand healthcare concerns without judgement, while simultaneously providing reassurance that solutions can be identified. Understanding issues such as participant mindset made patients feel that their healthcare plan would work for them, because it was made for them. Further underscoring why patient-provider relationships are so important, one participant stated

“*When I get down and out, she makes me feel better about the situation, that things will get better, and they have. I feel much better than I did when she first started calling me.*” (Madison, 67)

Above, the participant highlights how experiences with encouragement and reassurance during moments of doubt in their healthcare journey reinforced the notion that their diabetes management can improve. Together, these experiences reveal the importance of safe spaces (e.g., judgment-free, encouraging, and individualized) for generating prosocial patient-provider relationships.

### 3.2. Theme 2: Experiences with Diabetes Education and Knowledge Attainment

Participants consistently reported how their experiences with Diabetes Solutions prompted new ways of making meaning of their relationship with food in their everyday life. Many patients reflected on how their food decisions felt more manageable with a clearer understanding of nutrition, glucose management, and overall health.

“*I have better knowledge of how food breaks down in my body… a much better understanding of my body holistically.*” (Ashley, 30)

“*It changed the way I look at food and my relationship with food. I can still enjoy things but know my limits. It was a wonderful education because I just didn’t have the knowledge I needed [before Diabetes Solutions].*” (Samantha, 60)

The quotes above reflect shifts in how participants make meaning of the role of food in their everyday lives after their diabetes diagnosis. Samantha notes that they previously did not have the knowledge needed to manage their diabetes without entirely giving up aspects of their life that they enjoyed prior to their diabetes diagnosis. Their experiences with Diabetes Solutions gave them control over their relationship with food in a way that allowed them to continue finding joy in food in responsible ways.

Noting the importance of their experiences with education on diabetes management, one participant noted how the education they experienced with Diabetes Solutions made them feel like they had the tools to make actionable changes such as reading food labels.

“*Now I look at labels more. If I hadn’t gone through it, I would probably still be doing the same old stuff. I contribute my lifestyle changes to them.*” (Lauren, 55)

Ultimately, individualized experiences with Diabetes Solutions also enhanced food knowledge that helped participants regain control over their relationship with food in ways that prompted lifestyle changes in support of their diabetes management.

### 3.3. Theme 3: Experiences with Lifestyle Changes

Patients involved with Diabetes Solutions reported shifts to how and why they prioritized lifestyle changes related to their diabetes diagnosis. These shifts in meaning-making were often discussed in conversation with the individualized nature of their program. Patients reported how the conversations they experienced with their provider helped to reorient their behaviors related to their diabetes management around other important aspects of their life, such as grandchildren. This provided a new layer of meaning behind why behaviors such as weight loss and exercise were important to patients. Because of this, changes in diet, physical activity, and mindset were related to family and long-term health. One participant reflected:

“*She asked me, ‘Where do you want to be in 10 years?’ I have 10 grandchildren. I want to be active and part of their lives. I knew if I didn’t lose weight and start being more conscious, that might not happen.*” (Jessica, 67)

In the quote above, the participant reflects on how a desire to be active in the life of their grandchildren in 10 years prompted them to prioritize weight loss and conscientious decision-making related to their diabetes. Exercise was another common lifestyle change reported by participants.

“*I started exercising, because I knew it would help. My goal was to get down on the floor and play with my youngest grandchild, and that has happened. That’s the happiest thing for me.*” (Sarah, 70)

Participants often credited the nonjudgmental support of coaches with empowering them to take control of previously overwhelming habits. For Sarah, exercise itself was experienced differently because it was a way of ensuring longevity in her relationships with her grandchildren instead of solely a form of diabetes management. Ultimately, behaviors such as diet and exercise held new meaning for participants and were experienced differently after conversations with providers contextualizing the relative importance of diabetes management for other priorities in their lives.

### 3.4. Theme 4: Experiences with Quality of Life Changes

Participant experiences with *Diabetes* Solutions supported the notion among participants that diabetes is a manageable condition rather than a limiting burden, reinforcing the idea that participants could lead a largely normal life and maintain important aspects of their identity. As stated by one participant,

“*Being diabetic is not the end of the world. I can still have a fulfilling life, but I just have to do some things differently now. My quality of life does not have to decline because I’m diabetic.*” (Daniel, 55)

The above quote highlights how experiences with individualized programming helped to prevent participants from allowing diabetes to define their lives. Relatedly, other participants shared how they began feeling more confident in managing the diabetes diagnosis aspects of their everyday social life, which remained intact. In doing so, participants were able to maintain parts of their lives that promote joy and are central to their identity. One participant said,

“*I feel much more confident about managing diabetes. I can still go out with friends, have a drink, and make good choices. I can still be a human being and have fun.*” (Elizabeth, 56)

“*It has helped me change the way I live and has made me a better and healthier person.*” (Michael, 71)

Participants frequently highlighted how managing their diabetes enhanced aspects of their life such as keeping up with grandchildren, becoming more socially active, and feeling more comfortable with their health journey. In essence, participants in our sample learned to coexist with their diabetes diagnosis instead of allowing it to take over.

## 4. Discussion

This qualitative exploration of the Diabetes Solutions telehealth coaching program highlights how individualized, relationship-centered diabetes support can help rural and underserved adults with diabetes and prediabetes manage their diagnosis in practical and sustainable ways. Across interviews, participants described how experiences with personalized coaching helped to reframe the way patients interact with healthcare providers in ways that had real-life implications for the way individuals make meaning of their relationship with food, lifestyle changes, and living with diabetes. This study contributes to the literature by showing not only that participants found telehealth DSMES acceptable, but that they experienced the program as personally relevant, emotionally supportive, and practical enough to translate knowledge into daily action.

Participants’ accounts suggest that the Diabetes Solutions model functioned as more than a diabetes education and coaching program. For many, it served as a structured support system that helped them reinterpret diabetes as a manageable condition rather than an overwhelming or defining diagnosis. This is particularly important in rural settings, where limited access to diabetes education, transportation barriers, provider shortages, and social determinants of health can make sustained diabetes self-management more difficult. The findings demonstrate that when telehealth is delivered through consistent, individualized coaching, it can preserve the relational elements of care that patients often value most while reducing common access barriers.

### 4.1. Personalized Coaching and Participant Engagement

One of the strongest findings was the importance of personalized, nonjudgmental coaching for shaping the way individuals experience their diabetes diagnosis. Participants repeatedly contrasted the program with more generic or prescriptive approaches to diabetes management. Rather than receiving standardized advice, they described coaching that accounted for their preferences, routines, emotional needs, food relationships, and personal goals. This individualized approach appeared to promote trust, openness, and sustained engagement. The relational nature of the coaching was especially meaningful. Participants described coaches as encouragers, accountability partners, and trusted guides. This type of support may be particularly important for individuals managing a chronic disease that requires daily decision-making. Diabetes self-management often depends on repeated choices related to food, movement, medication adherence, monitoring, and coping with setbacks. The presence of a supportive coach helped participants feel less alone in those decisions and more capable of continuing after challenges. These findings align with patient-centered care literature emphasizing that trust, shared decision-making, and respect for individual context can improve engagement and self-efficacy [29].

### 4.2. Translating Knowledge into Action

Participants also emphasized how program experiences shifted their understanding of diabetes in practical and personally meaningful ways. Many described learning how food affects blood glucose, how to read nutrition labels, how to manage portions, and how to make realistic substitutions without feeling deprived. Importantly, participants did not describe knowledge as abstract information. Instead, they described education that changed how they shopped, cooked, ate, exercised, and thought about their health. This distinction is important. Many individuals with diabetes receive general instructions to “eat better,” “lose weight,” or “watch carbohydrates,” but may not receive the individualized education needed to apply those recommendations in daily life. In this study, participants described the coaching process as helping them understand why changes mattered and how to make those changes in ways that fit their lives. This suggests that the program’s effectiveness may be tied not simply to information delivery, but to knowledge translation: helping participants convert health information into sustainable behaviors.

The acquisition of practical knowledge, particularly around food, nutrition, and glucose management, was central to participants’ progress. Several individuals noted that this was their first meaningful education on how daily behaviors affect diabetes outcomes. This finding reinforces the importance of DSMES programs not only for improving health literacy but also for transforming abstract medical advice into actionable strategies [30]. Importantly, participants credited their new understanding with long-term lifestyle changes, such as reading labels, moderating portion sizes, and adopting exercise routines.

### 4.3. Lifestyle Change Rooted in Personal Meaning

Lifestyle changes were commonly connected to deeply personal motivations, especially family, independence, and long-term quality of life. Participants often framed their goals around being able to play with grandchildren, remain active, avoid future complications, or feel better in everyday life. These motivations appeared to make behavior change more meaningful and durable. This finding reinforces the importance of connecting diabetes self-management goals to participants’ values. While clinical outcomes such as A1c reduction are important, participants often experienced success in more personal terms: feeling more energetic, becoming more confident, participating more fully in family life, or realizing they could still enjoy social activities while managing diabetes. These patient-defined outcomes are important because they reflect how individuals experience the real-world impact of chronic disease management.

### 4.4. Quality of Life and Diabetes Management

An important experience shared among Diabetes Solutions participants was that experiences with diabetes no longer felt like a huge detriment to their quality of life. Several participants described a shift from fear, confusion, or discouragement toward hope, control, and empowerment. They no longer viewed diabetes as inevitably limiting their lives, but as a condition they could manage with the right tools and support. This psychosocial shift is a meaningful outcome. Diabetes management can be emotionally burdensome, and feelings of shame, frustration, or hopelessness may interfere with engagement. Participants’ reflections suggest that supportive coaching helped reduce this burden by normalizing setbacks, offering encouragement, and helping individuals build confidence over time. These findings highlight the importance of evaluating DSMES programs not only through biomedical indicators, but also through patient-reported outcomes such as confidence, self-efficacy, emotional well-being, and perceived quality of life.

### 4.5. Lifestyle Changes Anchored in Family Motivation

Lifestyle changes were frequently tied to relational motivations, such as the desire to remain active with children or grandchildren. This echoes literature suggesting that family engagement and social support are critical drivers of sustained behavior change in chronic disease management [31]. Encouraging participants to link personal health behaviors to broader relational goals may therefore enhance the durability of telehealth interventions.

### 4.6. Telehealth as a Rural Care Strategy

The findings also support the use of telehealth as a practical strategy for expanding access to DSMES in rural communities. Participants were able to receive individualized coaching without the added burden of travel, which is especially relevant in geographically dispersed areas with limited healthcare infrastructure. Importantly, participants did not describe telehealth as impersonal or inferior. Instead, many experienced it as convenient, accessible, and sufficiently relational when delivered through consistent contact with a trusted coach. These findings suggest that telehealth can be an effective delivery model when it is designed intentionally. Technology alone is unlikely to transform diabetes care. Rather, the value of telehealth in this program appeared to come from the combination of accessibility, personalization, consistency, and emotional support. For rural diabetes programs, this suggests that successful telehealth models should prioritize relationship-building, flexible communication options, individualized goal setting, and ongoing accountability.

### 4.7. Policy and Reimbursement Implications

The findings from this study can help promote rural health policy and payer coverage decisions. Participants described experiences with telehealth coaching as accessible, personalized, and relationship-based, features that are particularly important in rural areas where transportation barriers, workforce shortages, and limited availability of accredited DSMES programs constrain access [2,6]. While the present study is qualitative and not generalizable, there is a degree of transferability to similar communities [32]. This means that making individualized healthcare available in other rural towns may have similar benefits depending on context (e.g., meaningful demographic differences and language barriers). These results suggest that individualized telehealth DSMES models are not only acceptable to patients but also likely to be a practical strategy for narrowing rural diabetes disparities when integrated into routine care.

However, translating effective telehealth DSMES programs into routine practice requires sustainable financing. While the present study does not explicitly delve into financial barriers to diabetes healthcare, the present research does suggest that individuals in a rural Mississippi community did benefit in meaningful ways from access to healthcare made available via telehealth. Thus, coupling telehealth medical care in rural communities with reimbursement strategies has the potential to magnify the capacity for telehealth to reduce barriers to care both geographically and financially. Currently, in the United States (US), many barriers exist to consistent payment models and methods of reimbursement for DSMES services. While Medicare recognizes DSMES as a preventive benefit, it is governed by specific program and billing requirements, and most other insurance companies do not provide reimbursement for such services, which limits their existence outside of grant funding or self-pay. These reimbursement constraints have real consequences: they push programs toward short-term grant dependence and limit scale-up, even when patient outcomes and satisfaction are strong. More consistent coverage across Medicaid and commercial plans is also needed, as coverage requirements vary substantially by state and payer, contributing to fragmented access and uncertainty for program planning. Ongoing national advocacy around telehealth reimbursement parity for rural providers highlights the broader relevance of aligning payment policy with access goals.

### 4.8. Recommendations for Program Enhancement

Findings suggest several opportunities to strengthen the Diabetes Solutions model:

1. Peer support groups could build community, reduce isolation, and reinforce accountability.
2. Family-inclusive coaching may amplify motivation and create supportive home environments.
3. Continuous feedback mechanisms, such as brief surveys or session evaluations, could inform real-time program adjustments and enhance participant satisfaction.

Future program development should preserve the individualized and relational qualities that participants identified as central to their success. As telehealth programs expand, maintaining the human connection within care delivery will be essential. The findings from this study suggest that participants valued not only the convenience of telehealth but also the feeling that someone knew them, encouraged them, and helped them apply diabetes education in realistic ways.

This qualitative exploration of the Diabetes Solutions telehealth coaching program highlights the value of personalized support, practical education, and relational encouragement in improving diabetes self-management among rural Mississippi residents. Across 27 interviews, participants consistently described enhanced knowledge, meaningful lifestyle changes, and improved confidence in living with diabetes. These findings underscore the role of individualized telehealth interventions in addressing gaps in healthcare access and education for underserved populations. These findings suggest that Diabetes Solutions was associated with improvements in patient-reported knowledge, behavior changes, confidence, and quality of life but also led to significant improvements in objective health outcomes, reinforcing the program’s potential to transform diabetes care in rural and underserved populations.

## 5. Limitations

Several limitations should be considered when interpreting the qualitative findings. Because participants were not randomly sampled and the main goal was to capture the lived experiences of program participants, findings may be subject to selection bias. Individuals invited to participate may have been more engaged, more comfortable communicating their experiences, or more likely to have had favorable program experiences than those who were not invited or who declined participation (N = 3). Although efforts were made to include participants with a range of clinical responses, including those who did not experience the greatest A1c reductions or other health improvements, the sample may still underrepresent participants with lower engagement, less favorable experiences, or limited ability to participate in an in-depth interview.

Additionally, because eligibility required completion of at least four coaching sessions and at least two A1c measurements, the findings primarily reflect the perspectives of participants with adequate intervention exposure and available follow-up data. As a result, the findings may not fully capture the experiences of participants who discontinued participation earlier or faced barriers to continued participation. It should be noted that the goal of the study is to capture the lived experiences of program participants. Thus, while there are limitations to a sample containing the most engaged program participants, this sample can provide rich insights into experiences with program participation. Future research should explore barriers to program participation, which would lend itself to a more nuanced understanding of how experiences with telehealth programming for diabetes management.

The majority of participants (92.5%) who completed the interviews had Type 2 Diabetes and 7.5% had prediabetes, representing 25 and 2 participants, respectively. Thus, readers should be responsible when taking the findings of this study and considering how they apply to individuals with Type 1 Diabetes, Gestational Diabetes, or Latent Autoimmune Diabetes in Adults (LADA). While the majority of people with Diabetes in Mississippi and internationally have Type 2 Diabetes or prediabetes, it still warrants further exploration among the Type 1, pregnant, and LADA populations to determine experiences with coaching support for disease management and health outcomes.

Finally, because data were collected through retrospective qualitative interviews, responses may have been influenced by recall bias. Interviewer bias is also possible, although efforts were made to use a consistent interview guide and to support consistency across interviews. Structured interviews, while useful for consistency, have the potential to limit the depth of exploration into unanticipated insights. Further, the use of coding cycles can be considered a more positivist approach to a phenomenological study. These were intentional methodological choices to ensure focus during interviews and to lend a degree of structure to the identification of lived experiences among a larger sample [10]. Future research should explore these experiences with more in-depth interviews among a smaller sample of individuals to provide a more rich and detailed explanation of lived experiences. The sample included only Black and White participants, with limited demographic diversity, which constrains generalizability. Finally, the rural Mississippi setting provides important context but may not reflect experiences in more urban or resource-rich environments.

## 6. Future Research

Future studies should compare telehealth DSMES programs with in-person models to evaluate relative effectiveness. Research examining the role of family involvement, peer support, and technology access in shaping outcomes would also be valuable. Research examining the delivery of the intervention among youth, adults with Type 1 Diabetes, Gestational Diabetes, and the LADA population is warranted. Additionally, exploring reasons for program disengagement could inform strategies to reduce attrition and expand reach.

## 7. Conclusions

This study demonstrates that individualized telehealth DSMES appears to be a promising approach to diabetes support for rural and underserved populations. Participants in the Diabetes Solutions program described gaining practical knowledge, making sustainable lifestyle changes, improving confidence, and experiencing a better quality of life. Their narratives suggest that the program’s impact was rooted in more than convenience or access alone. The combination of personalized coaching, consistent encouragement, practical education, and accountability was described by participants as a shift from uncertainty and frustration toward confidence and self-efficacy.

The findings also underscore the importance of listening to patient voices when evaluating chronic disease programs. For participants, success was not defined only by clinical measures, but also by being able to understand their bodies, make informed food choices, remain active with family, participate in social life, and feel hopeful about the future. These outcomes reflect the broader purpose of diabetes self-management support: helping individuals live well while managing a complex chronic condition. In rural communities where barriers to diabetes education and specialty support remain substantial, telehealth coaching offers a promising strategy for delivering accessible, relationship-centered care. However, the findings suggest that the strength of this model lies not simply in the use of telehealth, but in how telehealth is used.

This study provides evidence that telehealth DSMES programs, when built on personalized coaching and consistent encouragement, may support improvements in diabetes self-management in rural and underserved populations. Participants in the Diabetes Solutions program reported increased knowledge, sustainable lifestyle changes, and improved quality of life. These findings support the potential of telehealth to bridge gaps in healthcare access while supporting relational, educational, and emotional dimensions of chronic disease management.

At the same time, the sustainability of effective telehealth DSMES programs remains constrained by reimbursement and coverage gaps. A clear call to action emerges from these results: policymakers and payers should modernize reimbursement pathways so that evidence-based telehealth DSMES can be implemented broadly in rural settings and sustained as part of routine chronic disease care. Aligning payment policy with demonstrated patient benefit is a necessary step toward durable, equitable improvements in diabetes outcomes across rural communities.

Overall, this study contributes to the growing evidence that telehealth-based DSMES can support meaningful improvements in diabetes self-management and quality of life. Future efforts should continue to evaluate clinical outcomes, explore strategies such as peer and family support, and examine how this model can be adapted for diverse rural populations. Most importantly, diabetes care models should preserve the relational and educational elements that participants in this study identified as transformative. Programs that are individualized, flexible, supportive, and grounded in patient goals may be especially well positioned to improve engagement and promote lasting behavior change.

## Figures and Tables

**Table 1:** Demographic characteristics of interview participants (N = 27).

Characteristic	N	%
Age Group		
20–39 years	5	18.5
40–59 years	7	25.9
60–79 years	15	55.6
Gender		
Female	21	77.7
Male	6	22.2
Race		
White	16	59.3
Black	11	40.7

## Data Availability

The original contributions presented in this study are included in the article. Further inquiries can be directed to the corresponding author.

## Abbreviations

A1c	Hemoglobin A1c
CDCES	Certified Diabetes Care and Education Specialist
DSMES	Diabetes Self-Management, Education, and Support
DSMT	Diabetes Self-Management Training (Medicare’s term for DSMES)
FQHC	Federally Qualified Health Center
HCPCS	Healthcare Common Procedure Coding System
PPS	Prospective Payment System
RHC	Rural Health Clinic
US	United States

## Appendix A

**Table A1:** Interview questions.

Question #	Interview Question
1	What is your age, race, and gender?
2	What made you want to enroll in Diabetes Solutions?
3	Tell me about your experience with the program and how long you received coaching.
4	Did you use phone call, Zoom, or facetime, and are you satisfied with this telehealth approach?
5	How has Diabetes Solutions coaching contributed to your understanding and management of diabetes?
6	How have your health indicators increased, decreased, or stayed the same since starting the program? (blood sugar levels, A1C, weight)
7	What are you most proud of as far as how your health has improved since enrolling in the program?
8	In terms of lifestyle changes, such as diet and physical activity, how has the program supported or guided you in making sustainable changes?
9	The program covers a lot of ground. Can you identify one piece of advice or knowledge you’ve gained that has had the most significant impact on your daily diabetes management?
10	How has the program’s approach to personalized coaching and support helped in addressing your unique needs and concerns related to diabetes?
11	If you’ve tried things in the past, what made this feel different that empowered you to achieve success?
12	Looking at your journey with the program, how do you feel about your ability to manage your diabetes now versus when you first started?
13	Is there any you did not like, or you wish you could change/improve about the program?
14	Finally, what would you say to someone who is considering joining this program but is hesitant or unsure about its effectiveness? Would you recommend it and why?

Note: “#”: # refers to the question number.
